# Alteration of Porcine Intestinal Microbiota in Response to Dietary Manno-Oligosaccharide Supplementation

**DOI:** 10.3389/fmicb.2021.811272

**Published:** 2022-02-10

**Authors:** En Yu, Daiwen Chen, Bing Yu, Zhiqing Huang, Xiangbing Mao, Ping Zheng, Yuheng Luo, Heng Yin, Jie Yu, Junqiu Luo, Hui Yan, Jun He

**Affiliations:** ^1^Institute of Animal Nutrition, Sichuan Agricultural University, Chengdu, China; ^2^Key Laboratory of Animal Disease-Resistant Nutrition, Chengdu, China; ^3^Dalian Institute of Chemical Physics, Chinese Academy of Sciences, Dalian, China

**Keywords:** manno-oligosaccharide, weaned pigs, intestinal microbiota, microbial metabolites, intestinal health

## Abstract

Manno-oligosaccharide (MOS) is a prebiotic derived from natural plants or yeasts. Here, we explored the response of intestinal microbiota and epithelial functions after ingestion of MOS in a porcine model. Sixteen pigs were randomly assigned into two treatments and fed with basal or MOS-containing (0.3% MOS) diet for 21 days. Results showed that MOS supplementation increased the cecal acetate content and ileal 16S rRNA gene copies (*p* < 0.05). Importantly, MOS decreased the abundance of phylum Proteobacteria in cecal digesta (*p* < 0.05). Moreover, MOS elevated the expression level of SCL5A8 and GPR109A but decreased the expression levels of HDAC1 and TNF-α in the ileal and cecal mucosa (*p* < 0.05). MOS upregulated the expression levels of tight-junction protein (ZO-1, claudin-1, and occludin) and IGF-1 in the ileum and cecum (*p* < 0.05). This study presents the alteration of intestinal microbiota composition and intestinal barrier function after MOS administration, and facilitates our understanding of the mechanisms behind the dietary MOS-modulated intestinal microbiota and health.

## Introduction

Oligosaccharides are composed of monosaccharide units (2–20) with low molecular weight and low degree of polymerization, which have been looked as prebiotics because of their beneficial effects on intestinal microbiota (Delzenne, 2003; Mussatto and Mancilha, 2007). Oligosaccharides are resistant to digestion in the upper intestinal tract, but can be fermented by certain microorganisms in large bowel to produce short-chain fatty acids (SCFAs) (Garro et al., 2004; Mussatto and Mancilha, 2007; Quintero-Villegas, 2014). A previous study indicated that oligosaccharide-derived SCFAs not only promoted proliferation and differentiation of the intestinal epithelial cells (Blottier et al., 1999) but also promoted the growth of beneficial microorganisms such as *Bifidobacterium* and *Lactobacillus* species (He et al., 2021). Manno-oligosaccharide (MOS) is a non-digestible oligosaccharide isolated from sugar polymers present in the cell wall of yeast, which is composed of glucose and mannose units through β-1,4 glycosidic bonds (Yu et al., 2020). Owing to MOS’s special structures similar to the surface of intestinal mucosal (mannose), it may act as a guardian in the intestinal tract under subclinical infection (PC et al., 2000; Miguel et al., 2004). The health benefits of oligosaccharides have long been appreciated, and a diet containing MOS has been reported to increase the abundance of beneficial bacteria, enhance individual immunity, and maintain the intestinal epithelium integrity of poultry and pigs (Baurhoo et al., 2007; Castillo et al., 2008; Che et al., 2011).

Intestinal microbiota plays a vital role in maintaining intestinal homeostasis of humans and animals (Ma et al., 2017; Shi et al., 2017). However, the gastrointestinal tract of the fetus is sterile; therefore, early colonization of the infant gastrointestinal tract is crucial for the overall health of the infant (Wall et al., 2009; O’Toole and Claesson, 2010). Besides, intestinal microbiota is dynamic and easily affected by various factors (physiological and environmental); dietary strategies are effective approaches to modulate the composition of the intestinal microbiota for maintaining host health (Wan et al., 2020). A study revealed that dietary supplementation with prebiotics has a positive effect on enteric microbiota by selectively promoting growth of beneficial bacteria (Wall et al., 2009).

This study aimed to investigate the alteration of porcine intestinal microbiota in response to dietary MOS supplementation in weaned pigs. Pig is one of the excellent used model animals in biomedical studies, which has high similarity with humans involved in anatomy, genetics, and physiology (Meurens et al., 2011). Our study will be helpful to understand the mechanisms underlying the positive effect of MOS on modulating gut health.

## Materials and Methods

Studies involving animals were conducted according to the Regulations for the Administration of Affairs Concerning Experimental Animals (Ministry of Science and Technology, China, revised in June 2004). Sample collection was approved by the Institutional Animal Care and Use Committee of Sichuan Agricultural University, Sichuan, China (No. 20181105).

### Animal Housing and Sample Collection

Sixteen crossbred (Duroc × Landrace × Yorkshire) weaned pigs with an average initial body weight of 6.48 ± 0.14 kg were randomly allocated to two groups (*n* = 8). Pigs were kept individually and fed with a basal diet (BD) or BD containing 0.3% MOS. The diets (Supplementary Table 1) were formulated to meet the nutrient recommendations of the National Research Council 2012, and the chemical composition of the diet was analyzed using the AOAC method. Pigs were fed *ad libitum* and given free access to water. After 21 days, pigs were sacrificed by exsanguination under deep anesthesia *via* intravenous injection of sodium pentobarbital (200 mg/kg BW), and the intestinal tissues and mucosa samples were collected immediately. In addition, approximately 4 g digesta from the middle section of the ileum and cecum was transferred into sterile tubes and immediately frozen at –80°C for analysis of the SCFA concentration and the bacterial community.

### Metabolite Concentrations in Colonic Contents

The SCFA (acetic acid, propionic acid, and butyric acid) concentrations were determined using a gas chromatograph system (VARIAN CP-3800, Varian, Palo Alto. CA, United States; capillary column 30 m × 0.32 mm × 0.25 μm film thickness) following the previous method (Franklin et al., 2002). After vortex, the digesta was centrifuged at 4°C for 10 min (12,000 × *g*), and the supernatant (1 ml) was then transferred into an Eppendorf tube (2 ml) and mixed with 0.2 ml metaphosphoric acid. After 30 min of incubation at 4°C, the tubes were centrifuged at 4°C for 10 min (12,000 × *g*) and aliquots of the supernatant (1 μl) were analyzed using the GC with a flame ionization detector and an oven temperature of 100–150°C. The polyethylene glycol column was operated with highly purified N_2_ as the carrier gas at 1.8 ml/min.

### Measurements of the Cecal Digesta pH Values

Immediately after the pigs were killed, approximately 5 g of digesta was collected into the ice-bathed sterile centrifugal tube, and then the pH value of each sample was determined using a PHS-3C pH meter (Shanghai, China).

### Analysis of the Bacterial Community

Nucleic acids were extracted from 0.5 g of digesta sample using the Stool DNA kit (TIANGEN, China) according to the manufacturer’s instructions. Quantitative Insights Into Microbial Ecology (QIIME) software package was used to analyze the diversity and composition of the bacterial community of these samples (Caporaso et al., 2010). PCR amplifications were used to amplify the V3–4 region of the 16S rRNA gene; the primer sequences used the 515F/806R primer set (341F: 5′-CCTAYGGGRBGCASCAG-3′; 806R: 5′-GGACTACNNGGGTATCTAAT-3′). The amplification procedures were based on a previously published protocol (Caporaso et al., 2011). Briefly, the PCR conditions used were 5 min at 95°C, 35 cycles of 30 s at 94°C, 30 s at 55°C, and 90 s at 72°C, followed by 10 min at 72°C. Amplification was carried out using a Verity Thermocycler (Applied Biosystems). The PCR products derived from amplification of specific 16S rRNA gene hypervariable regions were purified by electrophoretic separation on a 1.5% agarose gel and the use of a Wizard SV Gen PCR Clean-Up System (Promega), followed by a further purification step involving the Agencourt AMPure XP DNA purification beads (Beckman Coulter Genomics GmbH, Bernried, Germany) to remove primer dimers. The sequencing was conducted on an Illumina MiSeq 2000 platform (Personal Biotechnology, Shanghai).

Pairs of reads from the original DNA fragments were merged using Fast Length Adjustment of Short reads (FLASH) (Magoč and Salzberg, 2011), which can quickly and accurately merge the paired-end reads. Sequencing reads were assigned to each sample based on the unique barcode. Sequences were analyzed with the QIIME software package and UPARSE pipeline (Edgar, 2013), in addition to custom Perl scripts to analyze alpha (within sample) and beta (between sample) diversity. UPARSE was used to pick operational taxonomic units (OTUs). Sequences were assigned to OTUs using 97% species-level sequence identity. The first sequence assigned to each OTU was used as the reference sequence for that OTU field. RDP classifier was used to assign taxonomic data to each representative sequence (Wang et al., 2007). Rarefaction curves were generated by using QIIME, and this software was also used to calculate both weighted and unweighted UniFrac for principal coordinate analysis (PCoA) and unweighted pair group method with arithmetic mean (UPGMA) clustering.

### RNA Isolation, Reverse Transcription, and Real-Time Quantitative PCR

The frozen intestinal mucosa samples (about 0.1 g) were ground in liquid nitrogen and homogenized in 1 ml of RNAiso Plus (Takara Biotechnology Co., Ltd., Dalian, China) to extract total RNA following the manufacturer’s instructions, and the purity and concentration of total RNA were detected by using a spectrophotometer (NanoDrop 2000; Thermo Fisher Scientific, Inc., Waltham, MA, United States); samples in which OD_260_/OD_280_ ratio ranged from 1.8 to 2.0 were deemed appropriate. Subsequently, a volume equivalent to 1 μg total RNA from each duodenal, jejunal, and ileal sample was used for reverse transcription into cDNA, which is based on the protocol of PrimeScript RT reagent kit with gDNA Eraser (Takara Biotechnology Co., Ltd.). This process consists of two steps: I, 37°C for 15 min; II, 85°C for 5 s.

The expression level of the target gene [G protein-coupled receptor 41 (GPR41), GPR43, GPR109A, sodium-couple monocarboxylate transporter 1 (SLC5A8), histone deacetylase 1 (HDAC1), HDAC2, HDAC3, IL-1β, IL-10, TNF-α, NF-κB, ZO-1, zonula occludens-1, occludin, claudin-1, insulin-like growth factor (IGF-1), glucagon-like peptide (GLP-2), epidermal growth factor (EGF)] in intestinal mucosa was quantified using q-PCR, and the oligonucleotide primer sequences used in qPCR are presented in Supplementary Table 2. qPCR was performed with the SYBR Green PCR I PCR reagents (Takara Bio Inc., Dalian, China) using a CFX96 Real-Time PCR Detection System (Bio-Rad Laboratories, Hercules, CA, United States). All cDNA samples were detected in triplicate. The reaction mixture (10 μl) contained 5 μl SYBR Premix Ex Taq II (Tli RNaseH Plus), 0.5 μl forward primer, 0.5 μl reverse primer, 1 μl cDNA, and 3 μl RNase-free water. The protocol used in qPCR was as follows: 95°C for 30 s, followed by 40 cycles at 95°C for 5 s and 58°C for 34 s. The generated gene-specific amplification products were confirmed by melting curve analysis after each real-time quantitative PCR assay. The housekeeping gene β-actin was used to standardize the mRNA expression level of target genes, which was calculated based on the 2^–ΔΔCt^ method (Fleige et al., 2006).

## Results

### Effect of Dietary Manno-Oligosaccharide Supplementation on Growth Performance and Microbial Metabolites

**FIGURE 1:**
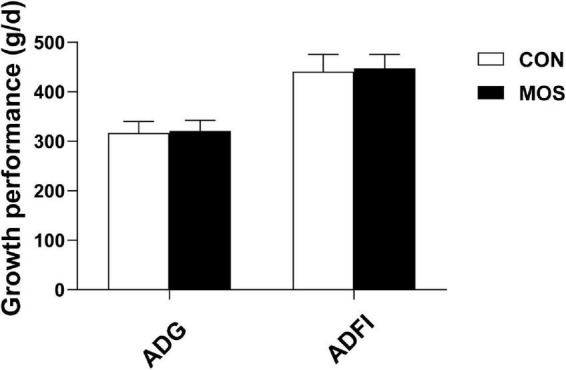
Effects of MOS on growth performance of weaned pigs. ADG means average daily gain; ADFI means average daily feed intake.

**FIGURE 2:**
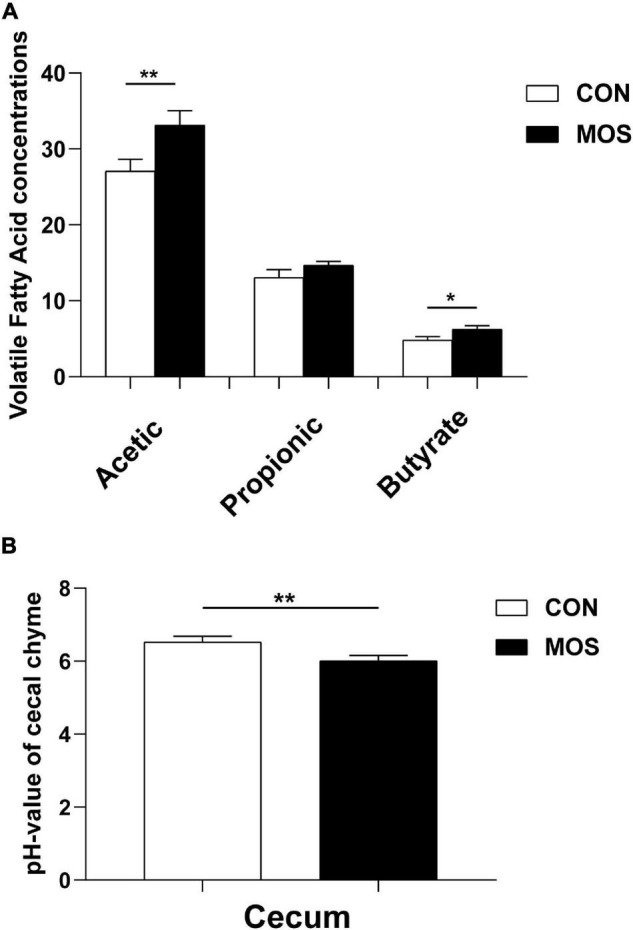
Effects of MOS on microbial metabolites (VFA) **(A)** and pH values **(B)** of cecal digesta. Digesta samples were collected from pigs fed with the basal diet (CON) or a basal diet supplemented with 3 g/kg MOS (MOS) (*n* = 8). **p < 0.05, *0.05 < *p* < 0.1.

Figure 1 shows that MOS has no effect on growth performance of weaned pigs, and Figure 2 reveals the effects of MOS supplementation on the SCFA content (A) and pH value of cecal digesta. Dietary MOS supplementation significantly increased the acetate content in cecal digesta compared with CON group (*p* < 0.05). Besides, MOS tended to increase the butyrate content in the cecal digesta (*p* > 0.05). Moreover, MOS significantly decreased the pH value (Figure 2) in the cecal digesta (*p* < 0.05).

### Effect of Dietary Manno-Oligosaccharide Supplementation on Total 16S rRNA Gene Copies in the Ileum and Cecum

**FIGURE 3:**
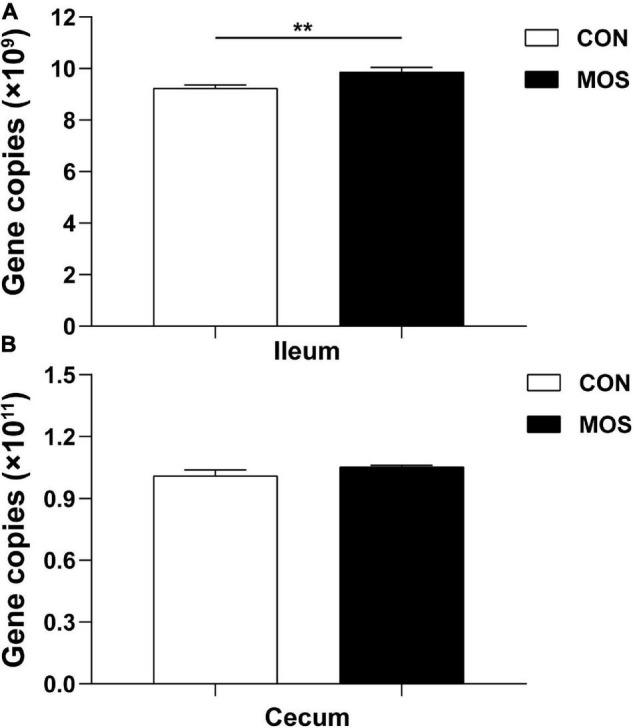
Quantitative PCR analysis of 16S rRNA gene copy numbers in the intestinal digesta. Ileum **(A)** and cecum **(B)** digesta samples were collected from pigs fed with the basal diet (CON) or a basal diet supplemented with 3 g/kg MOS (MOS) (*n* = 8). **p < 0.05.

The 16S rRNA gene copies were determined using quantitative PCR, and equal volumes of the purified DNA of all samples were used in the assay. Figure 3 shows that the average copy number in the cecal samples was higher than in the ileal samples. Dietary MOS supplementation had no significant influence on the total 16S rRNA gene copies in the cecal digesta. However, MOS significantly elevated the total 16S rRNA gene copies in the ileal digesta (*p* < 0.05).

### Effect of Dietary Manno-Oligosaccharide Supplementation on Bacterial Community Structures

**FIGURE 4:**
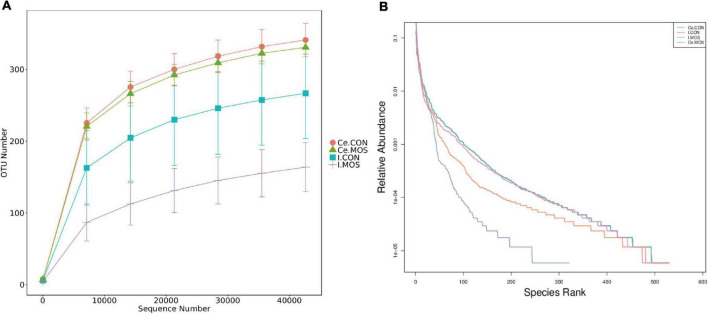
Rarefaction **(A)** and rank–abundance **(B)** curves. I.CON means ileal digesta samples from pigs fed with the basal diet. I.MOS means ileal digesta samples from pigs fed with a basal diet supplemented with 3 g/kg MOS; Ce.CON means cecal digesta samples from pigs fed with the basal diet; Ce.MOS means cecal digesta samples from pigs fed with a basal diet supplemented with 3 g/kg MOS (*n* = 4).

**FIGURE 5:**
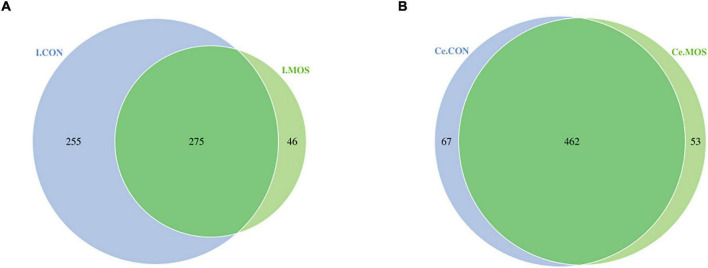
Number of identified OTUs in various comparisons. **(A)** Venn diagram shows various comparisons of ileal OTUs at the genus level; **(B)** Venn diagram shows various comparisons of cecal OTUs at the genus level. I.MOS means ileal digesta samples from pigs fed with a basal diet supplemented with 3 g/kg MOS; Ce.CON means cecal digesta samples from pigs fed with the basal diet; Ce.MOS means cecal digesta samples from pigs fed with a basal diet supplemented with 3 g/kg MOS. For 16S rRNA analysis, two digesta samples in each group were pooled (*n* = 4).

16S rRNA sequencing was used to compare digest samples based on the proportions of bacterial lineages in each specimen. Equal volumes of the purified DNA from each sample were amplified by PCR using bar-coded primers flanking the V3–4 region of the 16S rRNA gene, and samples were sequenced using the Illumina method. All sequencing information has been deposited in the National Center for Biotechnology Information (NCBI) and can be accessed in the Sequence Read Archive (SRA) under the accession number PRJNA768244. The rarefaction and rank–abundance curves directly indicated that the depth and the degree of evenness of sampling were adequate to assess the bacterial communities (Figures 4A,B). For the ileal samples, 46 OTUs were unique in the MOS group, whereas 255 OTUs were specifically identified in the CON group. A total of 275 OTUs were shared between two groups (Figure 5A). For cecal samples, 67 OTUs and 53 OTUs were specifically identified in the CON and MOS groups, respectively. Besides, the two groups shared 462 common OTUs (Figure 5B). In general, the number of OTUs identified in the cecum was higher than in the ileum.

**FIGURE 6:**
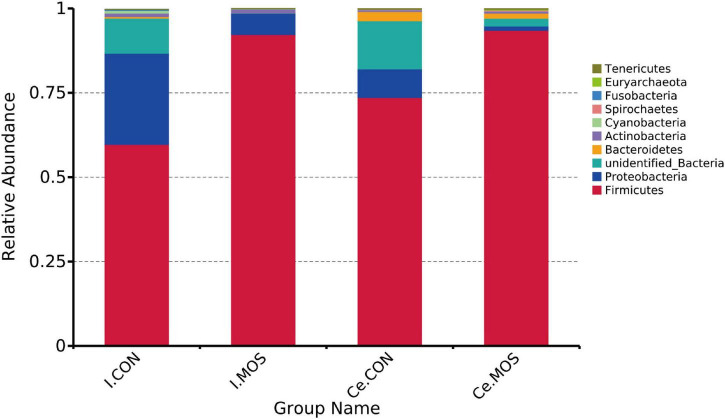
Bar graph shows the phylum level composition of bacteria. Color-coded bar plot shows the relative abundance of bacterial phyla across the different groups. I.MOS means ileal digesta samples from pigs fed with a basal diet supplemented with 3 g/kg MOS; Ce.CON means cecal digesta samples from pigs fed with the basal diet; Ce.MOS means cecal digesta samples from pigs fed with a basal diet supplemented with 3 g/kg MOS. For 16S rRNA analysis, two digesta samples in each group were pooled (*n* = 4).

**FIGURE 7:**
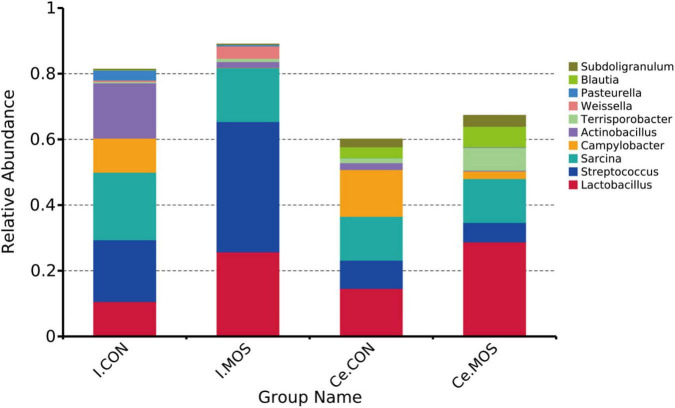
Bar graph shows the genus-level composition of bacteria. Color-coded bar plot shows the relative abundance of bacterial phyla across the different groups. I.MOS means ileal digesta samples from pigs fed with a basal diet supplemented with 3 g/kg MOS; Ce.CON means cecal digesta samples from pigs fed with the basal diet; Ce.MOS means cecal digesta samples from pigs fed with a basal diet supplemented with 3 g/kg MOS. For 16S rRNA analysis, two digesta samples in each group were pooled (*n* = 4).

We determined the similarity of microbiota communities by PCoA based on weighted UniFrac distance metrics (Supplementary Figure 1). The ileal microbiota from the MOS and CON groups was divided into disparate parts that were distinct in the PCoA. However, the cecal microbiota did not show significant differences between the two groups. In this study, all the qualified sequences from ileal and cecal samples were assigned to 18 and 16 known phyla, respectively. The relative abundant distribution of the top 10 intestinal bacterial at phylum and genus level between two groups of ileum and cecum are presented in Figures 6, 7. Firmicutes (92.28%) is the predominant phyla identified in the MOS group, whereas Proteobacteria (26.95%) is the predominant phyla identified in the CON group of ileal samples. Similarly, Firmicutes (93.54%) is the predominant phyla identified in the MOS group of cecal samples (Figure 6 and Supplementary Table 3). Compared with the CON group, MOS supplementation tends to elevate the abundance of Firmicutes (*p* > 0.05) in ileal and cecal samples, but significantly decreased the abundance of Proteobacteria (*p* < 0.05) in the cecal samples at phylum level. Furthermore, at genus level (Figure 7 and Supplementary Table 4), *Lactobacillus* is the predominant genera in ileal (25.78%) and cecal (28.80%) samples of MOS group. MOS supplementation tends to elevate the *Blautia* abundance in cecal samples but decreased *Actinobacillus* in ileal samples (*p* > 0.05).

**FIGURE 8:**
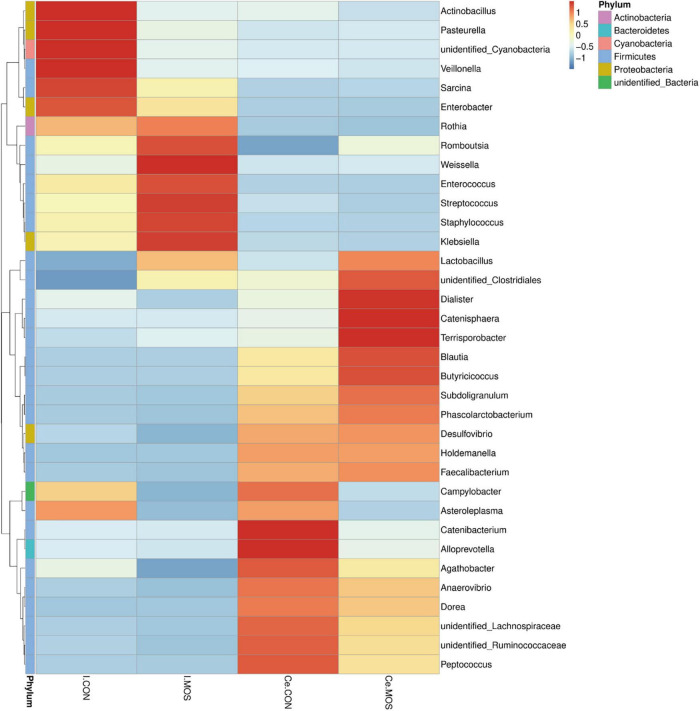
Heatmap distribution of OTUs at phylum level. OTUs were arranged in rows and are clustered on the vertical axis. Samples are arranged vertically and are on the horizontal axis. Different colors indicate the relative abundance of taxons. I.MOS means ileal digesta samples from pigs fed with a basal diet supplemented with 3 g/kg MOS; Ce.CON means cecal digesta samples from pigs fed with the basal diet; Ce.MOS means cecal digesta samples from pigs fed with a basal diet supplemented with 3 g/kg MOS. For 16S rRNA analysis, two digesta samples in each group were pooled (*n* = 4).

**FIGURE 9:**
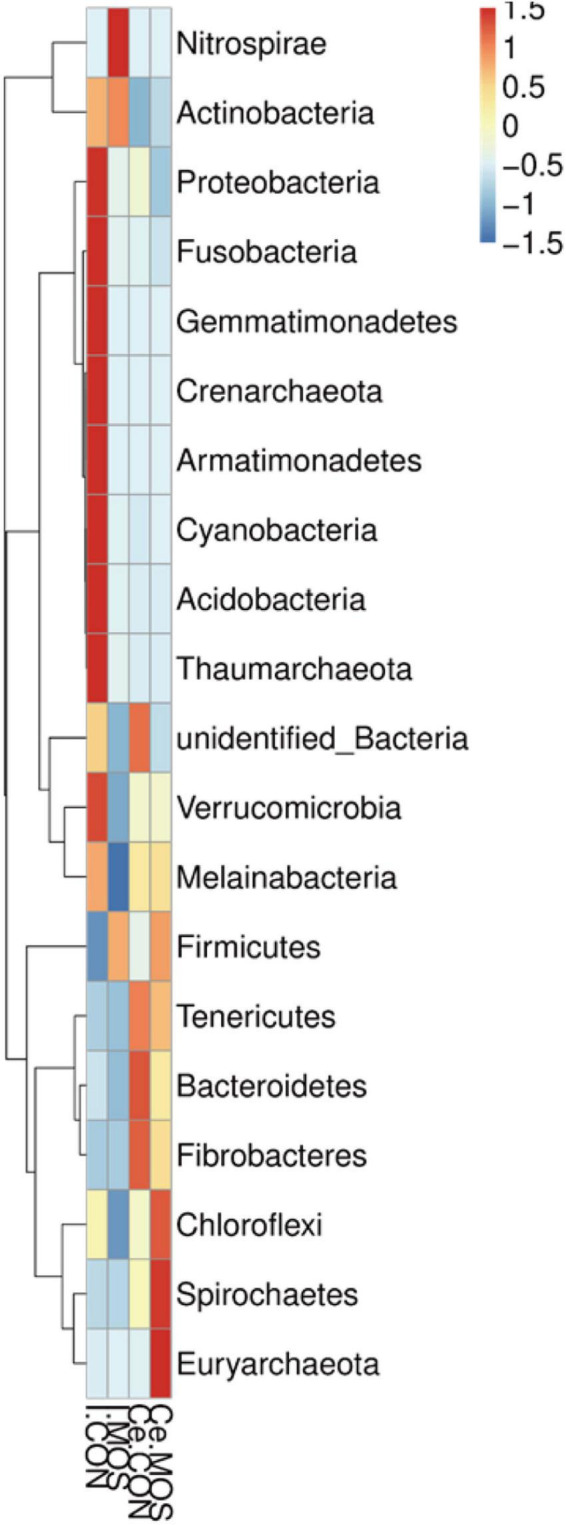
Heatmap distribution of OTUs at genus level. OTUs were arranged in rows and are clustered on the vertical axis. Samples are arranged vertically and are on the horizontal axis. Different colors indicate the relative abundance of taxons. I.MOS means ileal digesta samples from pigs fed with a basal diet supplemented with 3 g/kg MOS; Ce.CON means cecal digesta samples from pigs fed with the basal diet; Ce.MOS means cecal digesta samples from pigs fed with a basal diet supplemented with 3 g/kg MOS. For 16S rRNA analysis, two digesta samples in each group were pooled (*n* = 4).

All sequences filtered from the ileal and cecal samples were assigned to 35 known genera and 20 known phyla (Figures 8, 9 and Supplementary Tables 5, 6). The heatmap exhibits the abundance of the selected genera and phyla across the samples, which directly revealed the significant differences in the phylum distribution between the CON and MOS groups (Figures 8, 9). Figure 9 (Supplementary Table 6) shows that dietary MOS supplementation significantly decreased the abundance of cecal Proteobacteria at phylum level (*p* < 0.05), but tends to elevate the abundance of ileal and cecal Firmicutes (*p* > 0.05).

### Effect of Dietary Manno-Oligosaccharide Supplementation on Expression Levels of GPRs, HDACs, and Inflammatory-Related Genes

**FIGURE 10:**
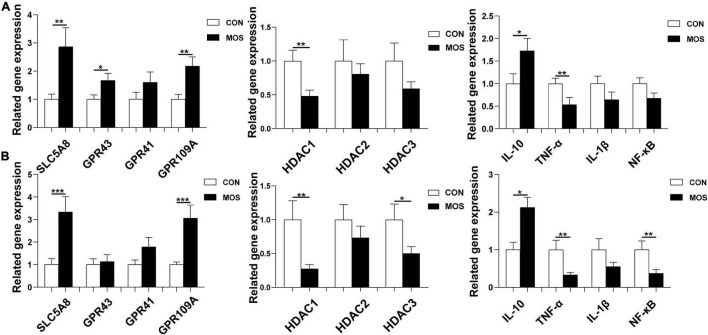
Effect of MOS on expression levels of GPRs, HDACs, and inflammatory cytokines. Ileum **(A)** and cecum **(B)** digesta samples were collected from pigs fed with the basal diet (CON) or a basal diet supplemented with 3 g/kg MOS (MOS) (*n* = 8). *0.05 < *p* < 0.1; **p < 0.05, ***p < 0.01.

As shown in Figure 10, dietary MOS supplementation significantly elevated the expression level of SCL5A8 and GPR109A in the ileal and cecal mucosa (*p* < 0.05). Nevertheless, MOS not only significantly decreased the expression levels of HDAC1 and TNF-α expression levels in the ileal mucosa (*p* < 0.05) but also downregulated HDAC1, TNF-α, and NF-κB expression level in cecal mucosa (*p* < 0.05). Moreover, MOS tended to elevate the expression level of IL-10 in the ileal and cecal mucosa (*p* > 0.05).

### Effect of Dietary Manno-Oligosaccharide Supplementation on Expression Levels of Critical Genes Related to Intestinal Barrier Functions

**FIGURE 11:**
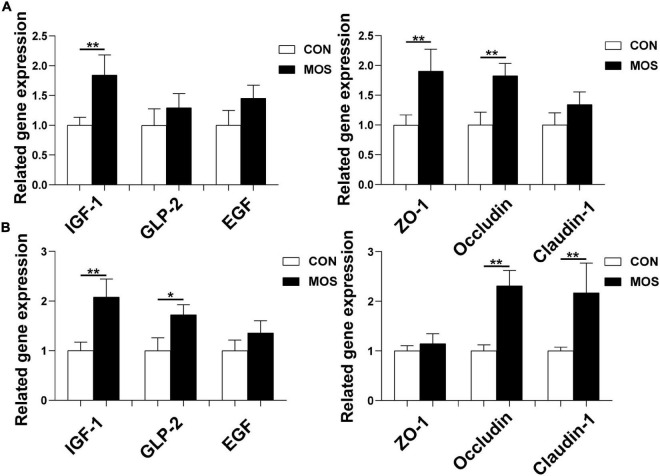
Effect of MOS on expression levels of genes related to intestinal barrier functions. Ileum **(A)** and cecum **(B)** digesta samples were collected from pigs fed with the basal diet (CON) or a basal diet supplemented with 3 g/kg MOS (MOS) (*n* = 8). *0.05 < *p* < 0.1, **p < 0.05.

As shown in Figure 11, dietary MOS supplementation significantly elevated the expression levels of tight-junction proteins (ZO-1, claudin-1, and occludin) in the ileum and cecum (*p* < 0.05). Besides, MOS significantly elevated the expression level of IGF-1 in the ileum and cecum (*p* < 0.05). Moreover, MOS supplementation tended to elevate the expression level of GLP-2 in cecum (*p* > 0.05).

## Discussion

Gut microbiota refers to the complex community of microorganisms residing in or passing through the GI tract, which plays an indispensable role in metabolic, physiological, and immunological processes in the body (Gerritsen et al., 2011). Previous studies have shown that oligosaccharides had dramatic influences on modulating the dysbiosis of the gut microbiota composition (Mitsuoka et al., 1987; Yang et al., 2008; Wan et al., 2020). In the present study, we explored the effect of dietary MOS on intestinal microbiota using a porcine model and found that MOS did not affect the growth performance of weaned pigs. SCFAs are the main end products of large bowel fermentation, which can serve as an energy source for intestinal epithelium and participate in regulation of cell growth, apoptosis, and various inflammatory responses (Havenaar, 2011; Lin et al., 2011; González-Herrera et al., 2019). Our results showed that dietary MOS supplementation elevated the SCFA content (acetate and butyrate) in the cecum. Previous studies have indicated the beneficial effect of SCFAs on improving glucose tolerance, maintaining the blood glucose homeostasis, and promoting the growth of probiotic bacteria (Puupponen-Pimiä et al., 2005; Yamashita et al., 2007; de Vadder et al., 2014). The elevated SCFA content is consistent with the decreased pH value in cecal digesta upon MOS supplementation. Both results indicated an antimicrobial potential of MOS in promoting the beneficial microbial fermentation in the intestine.

Previous studies suggested that dietary oligosaccharide supplementation significantly elevated the beneficial bacterial diversity in broiler chickens and weaned pigs (Baurhoo et al., 2007; Wan et al., 2020). In the present study, the 16S rRNA gene copy numbers were higher in the cecum than in the ileum, indicating that the large intestine is the main site of microbial fermentation. Interestingly, MOS significantly elevated the total copy numbers of the 16S rRNA gene in the ileum. A previous study indicated that the microbiota has the potential to regulate both the pro- and anti-inflammatory responses, and ecological imbalance in the intestinal flora may promote the development of various inflammatory bowel diseases (Round and Mazmanian, 2009). Firmicutes and Proteobacteria were the two dominant phyla identified in the ileum. The bacteria of phylum Firmicutes are involved in energy resorption (Chen et al., 2019), whereas the Proteobacteria are Gram-negative bacteria that includes numerous pathogenic species such as *Salmonella*, *Campylobacter*, and *Escherichia* (Patel and Pyrsopoulos, 2019). Importantly, high level of Proteobacteria may result in elevation of acetaldehyde, which increases intestinal permeability by disrupting the intestinal epithelium integrity (Arab and Martín-Mateos, 2017). Our result showed that MOS significantly decreased the abundance of Proteobacteria in the cecum, and tended to increase the abundance of Firmicutes in the ileum and cecum.

*Actinobacillus* species (belonging to the Proteobacteria phylum) are Gram-negative bacteria that can cause fatal pleuropneumonia in pigs (Rycroft and Garside, 2000). Our results showed that dietary MOS supplementation tended to decrease the abundance of *Actinobacillus* in the ileum. *Blautia* is a novel potential target/index against obesity and diabetes (Ozato et al., 2019). Moreover, *Blautia* is one of the most abundant genera in the intestine that can produce SCFAs (butyric acid and acetic acid) (Ozato et al., 2019). In the present study, MOS ingestion tended to elevate the abundance of *Blautia*. All these data suggested that MOS has a beneficial role of in regulating the intestinal microbiota.

Numerous studies indicated that SCFAs play an important role in maintaining gut health (Tan et al., 2014). A previous study indicated that SCFAs not only exert their effects by interacting with G-protein coupled receptors (e.g., FFAR2, FFAR3, OLFR78, and GPR109A) but also can serve as epigenetic regulators through inhibiting the histone deacetylase (HDAC) (Akhlaq, 2020). In the present study, we found that MOS ingestion significantly elevated the SLC5A8 and GPR109A expression level in the ileum and cecum. These results are consistent with the SCFA content, as the acetate and butyrate were found to activate the G protein-coupled receptors (SLC5A8 and GPR109A) (Singh et al., 2010). Interestingly, MOS supplementation decreased the HADC1 expression level, which is consistent with a previous report that butyrate and propionate not only inhibit HDAC but also can alter the expressions of specific genes *via* conformational changes in the active site of HDAC leading to its inactivation (Akhlaq, 2020). Furthermore, MOS decreased the expression levels of TNF-α in the ileum and cecum, indicating an anti-inflammatory potential of the oligosaccharides like with the MOS.

Tight junctions, consisting of cytoplasmic scaffold proteins such as ZO-1, claudins, and occluding, play a critical role in maintaining the intestinal barrier integrity and permeability (Citi and Cordenonsi, 1998; Yu et al., 2020). In the present study, the expression levels of occludin in the ileum and cecum were significantly elevated upon MOS supplementation. Moreover, MOS upregulated the expression of IGF-1 both in the ileum and cecum. IGF-I is a polypeptide hormone produced mainly by the liver in response to the endocrine GH stimulus, but it is also secreted by multiple tissues for autocrine/paracrine purposes (Puche and Castilla-Cortázar Larrea, 2012). Importantly, IGF-I is a trophic factor for the small intestine and exerts trophic effects on bowel mucosa, which showed beneficial effect in stimulating intestinal cell proliferate (Simmons et al., 1995).

## Conclusion

In summary, our study revealed an alteration of porcine intestinal microbiota in response to dietary MOS supplementation. The altered microbiota and SCFA content may contribute to the changes in inflammatory response and intestinal barrier functions *via* activating G protein-coupled receptor and enhancing the tight junction proteins. Our findings will be also helpful for the understanding of the mechanisms behind the dietary MOS modulating intestinal health.

## Data Availability Statement

The datasets presented in this study can be found in online repositories. The names of the repository/repositories and accession number(s) can be found in the article/Supplementary Material.

## Ethics Statement

The animal study was reviewed and approved by Institutional Animal Care and Use Committee of Sichuan Agricultural University, Sichuan, China (No.20181105).

## Author Contributions

JH conceived and designed the experiments. EY performed the experiments and wrote the manuscript. DC, BY, ZH, XM, PZ, YL, HY, JY, JL, and HY gave constructive comments for the results and discussion of the article. All authors have read and approved the final article.

## Conflict of Interest

The authors declare that the research was conducted in the absence of any commercial or financial relationships that could be construed as a potential conflict of interest.

## Publisher’s Note

All claims expressed in this article are solely those of the authors and do not necessarily represent those of their affiliated organizations, or those of the publisher, the editors and the reviewers. Any product that may be evaluated in this article, or claim that may be made by its manufacturer, is not guaranteed or endorsed by the publisher.
