# Supplementary figures and images for: Alteration of Porcine Intestinal Microbiota in Response to Dietary Manno-Oligosaccharide Supplementation
Source: Front Microbiol. 2022 Feb 10;12:811272. doi: 10.3389/fmicb.2021.811272 (PMC8866978; doi:10.3389/fmicb.2021.811272)

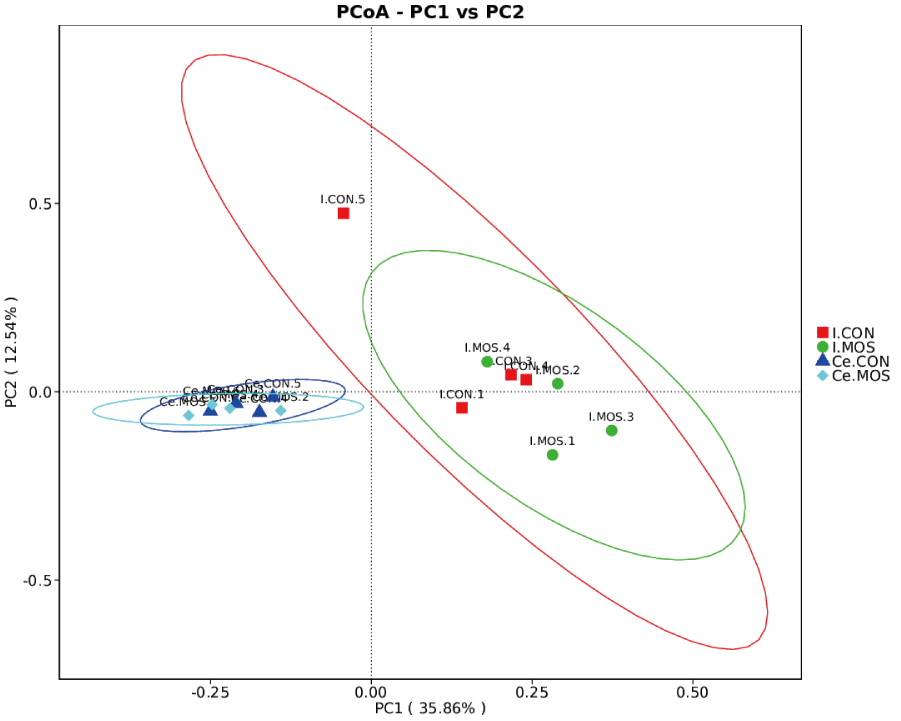

Supplement: Supplementary file 1 [file Image_1.TIF]
