# Supplementary material for: Alteration of Porcine Intestinal Microbiota in Response to Dietary Manno-Oligosaccharide Supplementation
Source: Front Microbiol. 2022 Feb 10;12:811272. doi: 10.3389/fmicb.2021.811272 (PMC8866978; doi:10.3389/fmicb.2021.811272)
Supplement: Supplementary file 2 [file Table_1.DOCX]

**Table S1. Experiment basal diet composition and nutrient level.**

| Ingredients | % | nutrient level | contents |
| --- | --- | --- | --- |
| Corn | 28.31 | Digestible energy (calculated, MJ/kg) | 14.78 |
| Extruded corn | 24.87 | Crude Protein (%) | 19.68 |
| Soybean meal | 8.50 | Calcium (%) | 0.81 |
| Extruded full-fat soybean | 10.30 | Available phosphorus (%) | 0.55 |
| Fish meal | 4.20 | Lysine | 1.35 |
| Whey powder | 7.00 | Methionine | 0.42 |
| Soybean protein concentrate | 8.00 | Methionine + cysteine | 0.60 |
| Soybean oil | 2.00 | Threonine | 0.79 |
| Sucrose | 4.00 | Tryptophan | 0.22 |
| Limestone | 0.90 |  |  |
| Dicalcium phosphate | 0.50 |  |  |
| NaCl | 0.30 |  |  |
| L -Lysine HCl (78%) | 0.47 |  |  |
| DL-Methionine | 0.15 |  |  |
| L -Threonine (98.5%) | 0.13 |  |  |
| Tryptophan (98%) | 0.03 |  |  |
| Chloride choline | 0.10 |  |  |
| Vitamin premix ^1^ | 0.04 |  |  |
| Mineral premix ^2^ | 0.20 |  |  |
| Total | 100 |  |  |

^1^The vitamin premix provided the following per kg of diet: 9000 IU of VA, 3000 IU of VD 3, 20 IU of VE, 3 mg of VK 3, 1.5 mg of VB1, 4 mg of VB 2, 3 mg of VB6, 0.02 mg of VB12, 30 mg of niacin, 15 mg of pantothenic acid, 0.75 mg of folic acid, and 0.1 mg of biotin. ^2^ The mineral premix provided the following per kg of diet: 100 mg Fe, 6 mg Cu, 100 mg Zn, 4 mg Mn, 0.30 mg I, 0.3 mg Se.
