# Supplementary material for: Alteration of Porcine Intestinal Microbiota in Response to Dietary Manno-Oligosaccharide Supplementation
Source: Front Microbiol. 2022 Feb 10;12:811272. doi: 10.3389/fmicb.2021.811272 (PMC8866978; doi:10.3389/fmicb.2021.811272)
Supplement: Supplementary file 3 [file Table_2.DOCX]

Table.S2 Sequences of primers for related genes of intestinal mucosa

| **ITEM** | **Primer and probe sequences (5′-3′)** | **Tm** | | **Product size (bp)** |
| --- | --- | --- | --- | --- |
| ZO-1 | CAGCCCCCGTACATGGGAGA | | 61.06 | 114 |
|  | GCGCAGACGGTGTTCATAGTT | | 61.27 |  |
| OCCLUDIN | CTACTCGTCCAACGGGAAAG | | 58.01 | 158 |
|  | ACGCCTCCAAGTTACCACTG | | 59.97 |  |
| CLAUDIN-1 | GCCACAGCAAGGTATGGTAAC | | 59.26 | 140 |
|  | AGTAGGGCACCTCCCAGAAG | | 60.62 |  |
| IL-10 | CGGCGCTGTCATCAATTTCTG | | 60.53 | 89 |
|  | CCCCTCTCTTGGAGCTTGCTA | | 61.24 |  |
| GPR109A | GGACAGCGGGCATCATCTC | | 60.60 | 200 |
|  | CCAGCGGAAGGCATCACAG | | 61.11 |  |
| SLC5A8 | CGCAGATTCCCTACTAACC | | 51.85 | 115 |
|  | GATTGTCAGTCCACCAT | | 51.73 |  |
| GPR-41 | TCTTCACCACCGTCTTATCTCAC | | 59.25 | 398 |
|  | CACAAGTCCTGCCCACCCTC | | 60.30 |  |
| GPR-43 | CTGCCTGGGATCGTCTGTG | | 60.15 | 249 |
|  | CATACCCTCGGCCTTCTGG | | 59.55 |  |
| HDAC1 | ACCTATGTTGATGCTGGGAG | | 56.98 | 124 |
|  | AAAGTAGTCGTTGTACGGAAG | | 55.70 |  |
| HDAC3 | TACTTTGCCCCCGGACTTCAC | | 59.96 | 185 |
|  | TCGTCAGTCCTGTCGTAGGT | | 59.96 |  |
| HDAC2 | ACAGGAGACTTGAGGGAT | | 53.86 | 232 |
|  | CACATTTAGCGTGACCTT | | 52.60 |  |
| GLP-2 | ACTCACAGGGCACGTTTACCA | | 61.92 | 150 |
|  | AGGTCCCTTCAGCATGTCTCT | | 60.55 |  |
| EGF | ATCTCAGGAATGGGAGTCAACC | | 59.49 | 166 |
|  | TCACTGGAGGATGGAATACAGC | | 59.56 |  |
| IGF-1 | CTGAGGAGCTGGAGATGTACT | | 60.69 | 137 |
|  | CCTGAACTCCCTCTACTTGTGTTC | | 60.56 |  |
